# Approach for Mandibular Reconstruction Using Vascularized Free Fibula Flap: A Review of the Literature

**DOI:** 10.7759/cureus.30161

**Published:** 2022-10-11

**Authors:** Bader Fatani, Jumana A Fatani, Omar A Fatani

**Affiliations:** 1 Dentistry, King Saud University, Riyadh, SAU; 2 Surgery, Specialized Medical Center Hospital, Riyadh, SAU; 3 Medicine, King Saud University Medical City, Riyadh, SAU

**Keywords:** implant, complication, techniques, free fibula flap, mandible reconstruction

## Abstract

Mandible reconstruction is one of the major challenges that face any maxillofacial surgeon worldwide. Different approaches and methods are used for mandible reconstruction, including pedicle flaps, titanium reconstruction plates, and bone grafts. A free fibular flap is used commonly and is considered the gold standard in mandibular reconstruction with a good success rate. Advantages of the free fibula flap include the long pedicle, flexible skin island, good length of dense cortical bone, consistent bone shape, minimal donor site morbidity, superior union rate, anti-collapse effectiveness, segmental blood supply, the potential for two skin paddles, and ease of harvest with a flap survival rate up to 95%. This current review aims to illustrate the approach for mandibular reconstruction using a vascularized free fibula flap.

## Introduction and background

Head and neck reconstruction has developed significantly throughout the years with the help of microvascular surgery and the ability to transfer the free tissue flap, which provided compatible soft tissue and bone from distant donor sites [1, 2]. Mandible reconstruction is one of the major challenges that face any maxillofacial surgeon worldwide [3]. Two main concerns need to be considered in association with mandible reconstruction: First, anatomical variation of the region. Second is the difficulty and complexity of mandibular movements [3]. Mandible reconstruction is required to reconstruct affected bone integrity after ablative surgery, infection, large jaw cysts, or trauma. Different approaches and methods are used for mandible reconstruction, including pedicle flaps, titanium reconstruction plates, and bone grafts [3]. However, the best method described for mandible reconstruction is the free osteocutaneous flap which is fixed by a titanium plate. The choice of which treatment method to be used depends on multiple factors, including patient factors, the skills of the medical team, patient tolerance to treatment, the requirement of occlusal rehabilitation, and the extension of the mandibular defect [3]. This current review aims to illustrate the approach for mandibular reconstruction using a vascularized free fibula flap.

## Review

Requirements for reconstruction 

The mandible can be reconstructed for different types of maxillofacial deficiencies such as traumatic defects, oncologic resections, congenital anomalies, osteoradionecrosis, significant atrophic jaws, or after jaw resection [1, 4, 5]. A multidisciplinary team approach for mandibular reconstruction is important. The aim of mandible reconstruction includes the re-establishing of form, improving quality of life, maximizing function, minimizing morbidity, and maintenance of integrity. Several factors need to be considered in patients requiring transfer of free tissue flap, including long-term prognosis, survival rate, patient expectations, reconstructive team, patient motivation, and patient surgeon and prosthodontist communication [1]. Many anatomic factors should be considered when evaluating reconstruction; these factors include the volume and location of the bone, the function of lips, pharyngeal, tongue, microvascular anastomosis and anatomy of the harvest distant tissue, status of dentition, maxillomandibular relationship, and mouth opening [1]. Oral hygiene, surgical steps, implant cost, and timing of the surgery should also be considered [1]. Adequate history and physical examination are critical to assess the candidate patient for the jaw in a day procedure. Relevant medical history should be reviewed, and any possible comorbidities that can alter the success of the treatment, which include autoimmune or vascular disease [6]. Assessment of soft tissue is the most important factor in selecting patients undergoing jaw-in-day surgery. Maxillofacial CT and a CT angiogram of the bilateral lower limb should be obtained [6]. Free fibula flap reconstruction is usually favored in dentate patients that require the reconstruction of long-span defects such as total or subtotal mandibulectomy. However, in the reconstruction of smaller mandibular defects, a simple bone graft is usually the treatment of choice [1].

Types of bone flap 

Numerous sites for harvesting vascularized free flaps for reconstruction have been illustrated in the literature; these donor sites include fibula, scapula, metatarsal bone, radial forearm, femur, ilium, and rib [1, 7]. The most common donor sites were the fibula, scapula, and ilium. These donor sites have advantages and disadvantages depending on location, volume and length of soft tissue and bone, type and extension of defect, and whether maxilla or mandible reconstruction is performed. Donor site flaps have different characteristics depending on the length of the vascular pedicle, quality and quantity, availability and length of bone, soft-tissue skin paddle, and the possibility of osteotomy [1]. Osteocutaneous free flaps are the most cost-effective, efficient, and reliable method for oromandibular reconstruction [8].

Fibula flap

**Figure 1:**
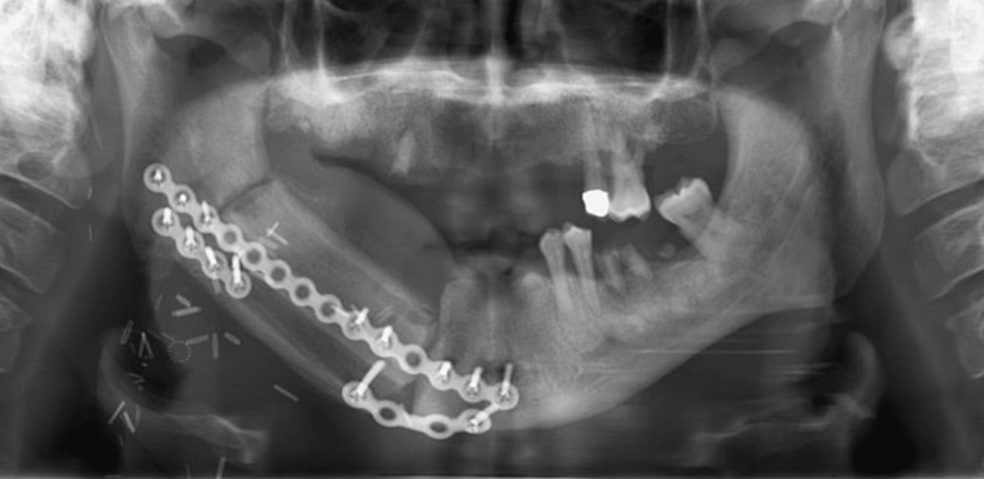
Double-barrel fibula free flap Source: Kokosis et al. [4] Image distributed under the Creative Commons Attribution Non-Commercial License.

The free fibula flap has become the technique of choice for mandibular and maxillary defect reconstruction in the last decade [9]. Fibular bone has the property of matching the jaws with its length structure which allows for the reconstruction of the maxilla and mandible after extensive bone resection. It can be harvested as a single flap, therefore permitting bulk replacement of bone and soft tissue [9]. A free fibular flap is commonly used and is considered the gold standard in mandibular reconstruction with a good success rate [4, 7, 8, 10-12]. A single free fibular flap can provide major support in reconstructing two jaws and a facial profile reconstruction with sufficient 3D spatial placement [10]. The fibula flap can reconstruct bony defects extending to 30 cm in length [2]. Free fibula flap has favorable bone quantity and quality, which can provide implantation rehabilitation of secondary teeth [6, 10]. Advantages of the free fibula flap include the long pedicle, flexible skin island, good length of dense cortical bone, consistent bone shape, minimal donor site morbidity, superior union rate and anti-collapse effectiveness, segmental blood supply, the potential for two skin paddles, and ease of harvest with a flap survival rate up to 95% [4, 12-15]. The vascularization of the free fibula flap helps in the preservation of the osteogenic potential and restores facial aesthetics, oral functions, and anatomic arch. However, the fragility of soft tissues, the thickness of subcutaneous tissues, thin cutaneous tissue, and the absence of a pelvilingual and vestibular groove can complicate the retention of dental prostheses [16]. The major disadvantage of the free fibula flap bone is the reduced vertical height. The typical height of the free fibula flap is usually 13 to 15 mm, which is proximate to the normal height of the atrophic edentulous mandible and almost half of the height of a dentate mandible [1]. A demonstration of a panoramic radiograph illustrating a double-barrel fibula free flap is shown in Figure 1.

Reconstruction methods

Fibula bone can have different structures depending on specific location and gender type [1]. The shape of the fibula bone is triangular at the head and converts to a more quadrilateral in the middle, then irregular or oval at the malleolus [1]. Distal fibula bone is used for a longer vascular pedicel length [1]. An ideal location should be obtained during the placement of fibula bone to ensure appropriate implant angulation placement and to avoid contact between the screws and the reconstruction plate [1]. Reconstruction plates with monocortical screws are an ideal choice compared with multiple miniplate fixations [1]. The harvesting of the free fibula flap should start after the identification of the neck vessels and adequate preparation of the recipient site. After neck dissection or irradiation, occasionally, it is difficult to locate recipient vessels in the neck. Thus, distant vessels or contralateral neck vessels may be required. Vein graft might increase the risk of subsequent flap loss and microvascular thrombosis [17]. A minimal number of screws should be considered with adequate stability. Inferior placement of the fibula will create a better facial appearance. However, due to the lack of alveolar height in adult dentate patients, a taller supra structure should be required. Thus, the fibula segment should be placed 5 to 10 mm above the inferior border of the mandible. A 10 to 15 mm space should be considered between the upper edge of the fibula and the occlusal plane of the opposing dentition to provide space for the implant framework and ensure an adequate emergence profile [1]. For endosseous implant placement, a minimum of 5 mm width and 10 mm height must be acquired in the fibula bone. Stabilization of segments should be done with plates and one to two monocortical screws to preserve the highest bone vascularity. For long fibula segments, at the very least, two monocortical screws must be used to maintain stabilization of the section, prevent micromovement at the osteotomy region, and avoid rotational forces of the segments [1]. Fibula flap reconstruction includes the position of 10 to 16 mm thick segments of soft tissues and bone. The harvested graft is thicker than the gingiva; these harvested grafts are not fixed by the periosteum to the bone and are not keratinized. In addition, they do not reconstruct the vestibule. Thus, vestibuloplasty and thinning of the flap are frequently needed [16].

Role of virtual surgical planning 

Virtual surgical planning before reconstruction using 3D technology has shown significant improvements in postoperative results, such as increased function, reduced operation duration, and increased aesthetics [4]. Preoperative virtual surgical planning is important and should be considered, especially in complex defects [4]. However, the disadvantages of these models are the high cost of these models and are not yet considered an integral part of the surgical approach [4]. The use of cutting templates, 3D printing models, and prebent plates are being used to increase the efforts to improve occlusal accuracy and establish operative efficiency during mandibular reconstruction [18]. Modern virtual technology and equipment help in the placement of guided implants and fibulas in restorative positions which allows for immediate dental rehabilitation [19]. Template design and computer modeling can increase the accuracy of fibula contouring and save time, mainly for surgeons with fewer skills and experience. However, it has a significant cost [8].

Soft-tissue management 

**Figure 2:**
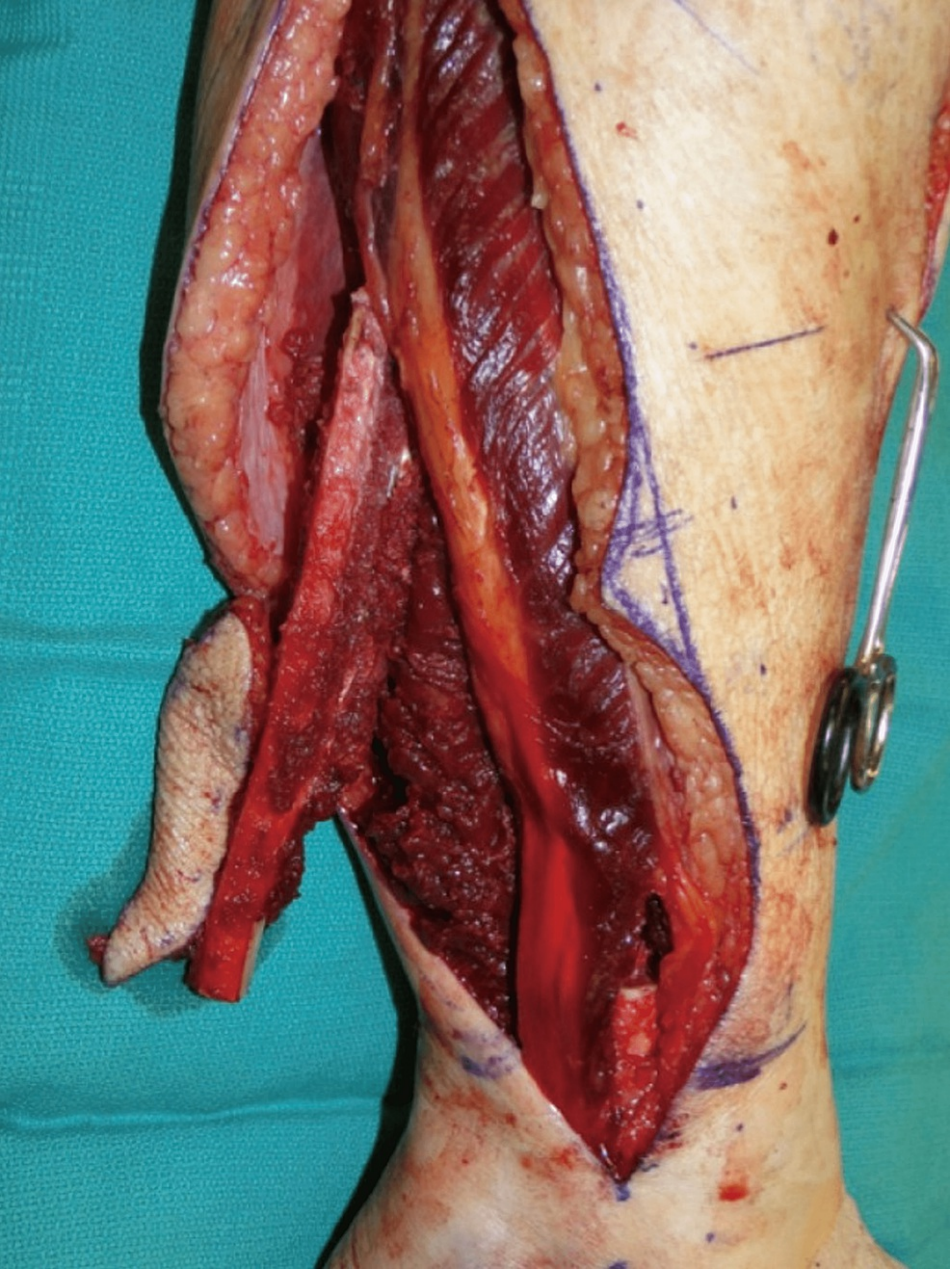
Osteoseptocutaneous fibula flap in situ Source: Kokosis et al. [4] Image distributed under the Creative Commons Attribution Non-Commercial License.

It's been demonstrated that oral characteristic strongly correlates with the size of soft tissue and no longer the dimensions of the mandibular bony defect. For the reconstruction of the soft-tissue defect, frequently a skin paddle is involved with the free fibula flap. This skin paddle delivers essential bulk and maintains integrity by splitting apart the oral cavity and other sites such as the nasal cavity, neck, and sinuses. Proper control of oral soft tissues is important for long-term stability, oral health, shape, and function [1]. According to a study that was done in Italy to compare traditional versus micro-invasive intraoral surgical approaches, the results showed no significant differences between the two groups in any of the features measured [11]. A demonstration of osteoseptocutaneous fibula flap in situ can be seen in Figure 2.

Implant placement considerations with fibula-free flap

Preoperative implant assessment should include the identification of patients who are motivated and willing to maintain their oral hygiene, follow instructions accurately, and undergo all surgical steps. Dental caries, reduced mouth opening, and periodontal disease should be reported before starting the implantation. The presence or absence of vestibular sulcus, the thickness of intraoral soft tissues, mobility of the tongue, oral functions, and efficiency of lip closure must also be checked [16]. Endosseous implants can generally be placed in a fibula-free flap by two mechanisms, primary or secondary. Primary can be placed at the time of fibula harvest immediately [1]. Secondary can be delayed by six to 12 months [1]. Primary implantation reduces rehabilitation time, especially in oncologic patients, and has a similar success rate as secondary implants. If delayed implant placement is favored, it must be placed at least six to 12 months following the graft once muscle healing and bone remodeling are finished to prevent implant failure arising from improper placement [16]. Survival and complication rates of these implants are reported to be similar with a similar safety profile. Single-stage complete reconstruction or prefabricated flaps, also known as jaw-in-a-day, have been reported. However, these are less commonly used [1]. The bicortical bone of the free fibula flap has an outstanding facility to receive dental implants, with the implant survival rates being as high as 93-99%. These prostheses are associated with important oral functions such as mastication, swallowing, and speech, with dental implants being a standard part of the rehabilitation plan for free fibula flap [13]. A previous study done in New York investigated the success of implants placed in fibula flaps, and results significantly revealed that delayed implant placement in free fibula flaps is highly successful [20]. During implanting at the same time as mandibular reconstruction, determination of the implant topography should be done after the bone graft is fixed temporarily. Placement of an implant at the same time as mandibular reconstruction is challenging; thus, soft tissues, implant placement, prosthetic result, and graft placement should all be taken into consideration [16]. A minimum of 10 mm height and 5 mm width is required in the fibula for placement of an endosseous implant [1].

Complications

Many complications have been reported after free fibula flap placement. However, these complications rarely occur [1, 13]. Mandible osteoradionecrosis (ORN) is a rare condition. However, it is considered a critical complication, especially after radiotherapy [21]. Marginal bone loss and peri-implantitis are the most common complications concerning implant placement in a free fibula flap. In addition, the most common cause of implant failure in free fibula flaps is peri-implantitis [1]. The author also reported fractures of the fibula, abscesses, flap necrosis, tumor recurrence, fracture or exposure of osteosynthesis, osteomyelitis, osteoradionecrosis, and devascularization of the fibula at the implant site [1, 13]. Moreover, oral cancer can occur around the implant and present as peri-implantitis. Tumor recurrence at the implant region occurs following primary implant placement. Tobacco, radiation dose, seeding of the implant placement bed, peri-implantitis, and chronic irritated oral mucosa were also reported to be associated with the recurrence at primary implant sites. Parallel to the long axis of the shaft, a linear fracture of the fibula could be present during implant placement in case the final osteotomy did not reach the apex. In this case, the fibula segment and implant can be saved by the maintenance of the fibula segment in place using bicortical lag-screw fixation [1]. Morbidity of the donor site was reported as mild, with no evidence of decreased lower limb performance. In addition, there were no reported functional limitations during gait and stair performance were found [9]. A single disadvantage of the free fibula graft was reported, which is the height discrepancy between the transplanted fibula and the native mandible, particularly in the anterior region [4, 16]. The complications of wound healing in donor areas are common early complications which include delayed healing, bleeding, and infection. However, these do not commonly cause long-term problems and do not affect the functional outcome [14].

## Conclusions

Mandibular defects are classified into extensive composite, composite, compound, and isolated bone defects. Mandible reconstruction is required to reconstruct affected bone integrity after ablative surgery, infection, large jaw cysts, or trauma. The mandible can be reconstructed for different types of maxillofacial deficiencies such as traumatic defects, oncologic resections, congenital anomalies, osteoradionecrosis, significantly atrophic jaws, or after jaw resection. A free fibular flap is commonly used and is considered the gold standard in mandibular reconstruction with a good success rate. Advantages of the free fibula flap include the long pedicle, flexible skin island, good length of dense cortical bone, consistent bone shape, minimal donor site morbidity, superior union rate and anti-collapse effectiveness, segmental blood supply, the potential for two skin paddles, and ease of harvest with a flap survival rate up to 95%. Preoperative virtual surgical planning is important and should be considered, especially in complex defects. However, the disadvantages of these models are the high cost of these models and are not yet considered an integral part of the surgical approach. Endosseous implants can generally be placed in a fibula-free flap by two mechanisms, primary or secondary. Primary can be placed at the time of fibula harvest immediately. Secondary can be delayed by six to 12 months. Primary implantation reduces rehabilitation time, especially in oncologic patients, and has a similar success rate as secondary implants. Marginal bone loss and peri-implantitis are the most common complications concerning implant placement in the free fibula flap. Authors also reported fractures of the fibula, abscesses, flap necrosis, tumor recurrence, fracture, or exposure to osteosynthesis, osteomyelitis, osteoradionecrosis, and devascularization of the fibula at the implant site.
